# A compact and high efficiency intracavity OPO based on periodically poled lithium niobate

**DOI:** 10.1038/s41598-021-84721-9

**Published:** 2021-03-03

**Authors:** Ke Wang, Mingyao Gao, Shuhui Yu, Jian Ning, Zhenda Xie, Xinjie Lv, Gang Zhao, Shining Zhu

**Affiliations:** 1 National Laboratory of Solid State Microstructures, School of Physics, School of Electronic Science and Engineering, College of Engineering and Applied Science, and Collaborative Innovation Center of Advanced Microstructures, Nanjing University, Nanjing, 210093 China; 2 Nanjing Star-Shining Technology Company Limited, Nanjing, 210043 China

**Keywords:** Mid-infrared photonics, Design, synthesis and processing, Applied optics, Lasers, LEDs and light sources

## Abstract

We demonstrate a compact, high efficiency and widely tunable intracavity singly resonant optical parametric oscillator (IC-OPO) based on multichannel periodically poled lithium niobate (PPLN). The IC-OPO is composed of 808 nm pump laser diode (LD), Nd:YVO_4_ laser and linear OPO. The continuous-wave (CW) mid-infrared (MIR) output laser is tunable from 2.25 to 4.79 μm. The maximum output power exceeds 1.08 W at 3.189 μm at 9.1 W LD pump power and the conversion efficiency is 11.88%. We also build up a prototype with volume of 145 × 85 × 42.5 mm^3^ and its total weight is less than 2 kg. The measured power stability is 1.3% Root Meat Square (RMS) for a 3 h duration under simulated high temperature conditions of 40 °C. RMS is 2.6% for a 4 h duration when simulated temperature is − 40 °C.

## Introduction

With recent progress in the fabrication of periodically-poled ferroelectric materials, quasi-phase-matching (QPM) technique is growing rapidly, which can be used in optical parametric oscillator (OPO)^1,2^. In nonlinear optics, LiNBO_3_ is one of important crystals with large nonlinear coefficient (d_33_ ~ 27 pm/v)^3^, especially in widely tunable MIR OPO. MIR is the atmospheric window, which means it’s good for remote sensing through air^4^. Meanwhile, absorption of many industrial polluted gas peaks at 2–5 μm such as CH_4_, C_3_H_8_, HCl, HF and so on. MIR laser can serve as an exhaust emission detector^5^. In addition, MIR laser is widely applied in martial and medical field. It’s important to produce compact and high quality (high-efficiency and widely-tunable) MIR laser. A number of mid-infrared continuous waves based on IC-OPO have been reported^6–19^: Carleton et al. reported a CW IC-OPO based on PPRbTiOAsO_4_ pumped by 1064 nm Nd:YVO_4_ laser. At 3 W of input diode-laser, the maximum power of 65 mW of idler at 3.52 μm was obtained, which corresponds to an optical-to-optical conversion of efficiency of 2.17%^6^. In 2008, an IC-OPO based on PPLN pumped by 1064 nm Nd:YVO_4_ laser was described, whose conversion efficiency was 1.82% from 808 nm to 3.86 μm^8^. A CW singly-resonant IC-OPO based on a Yb :KYW laser had conversion efficiency of 4.08% at ~ 3500 nm^11^. Researchers paid more and more attention on the broadband and compact performance of IC-OPO^12–17^. In 2017, Haiyong Zhu demonstrated a CW singly resonate IC-OPO based on PPLN pumped by a diode-pumped Nd:YVO_4_ laser at 1064 nm. The conversion efficiency was 10.2% with tunable span from 2.95 to 4.16 μm^18^. They broadened the span which was from 2.62 to 4.16 μm with idler power of 2.11 W in 2018^19^. However , most OPOs are built on optical tables which are not portable. Thus, it’s difficult for them to be practically used in moving vehicles based on the air, land or water^20–22^. Yichen Liu reported a CW OPO module, not including pump, with volume of 220 × 60 × 55 mm^3^ in 2018, which covered a tuning range of 2.42–2.93 μm and 3.14–3.45 μm^23^.

**Figure 1:**
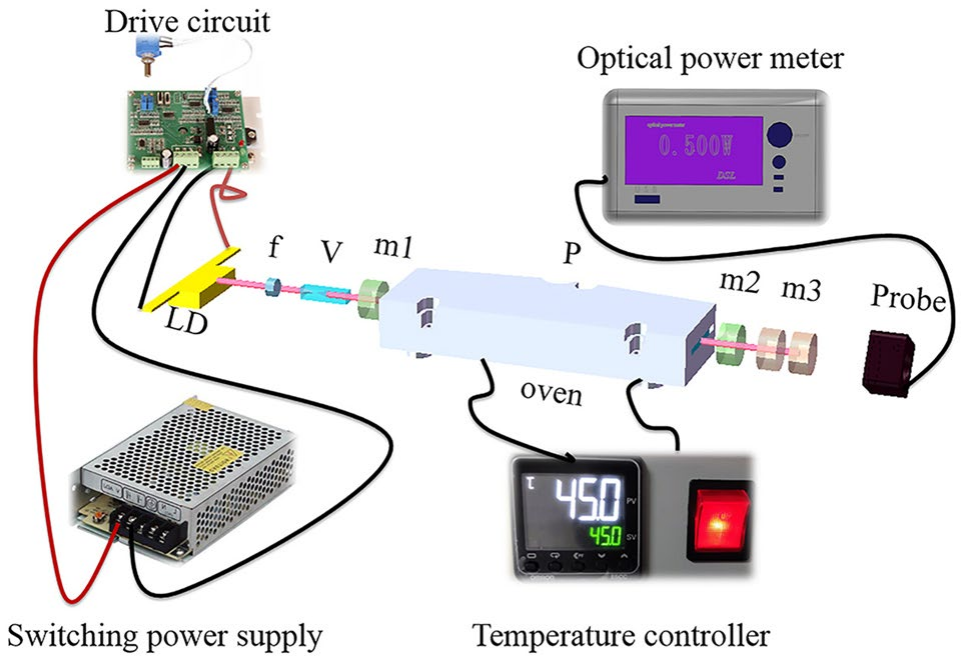
Experimental schematic diagram of IC-OPO. LD: solid 808 nm laser diode; f: focusing lens; V: Nd:YVO_4_ crystal; m1: IC-OPO input mirror; P: PPLN crystal; m2: IC-OPO output mirror; m3: dichroic mirrors (Solid Works 2018, https://www.solidworks.com/, Microsoft Office Power Point 2007, https://www.office.com/, Adobe Photoshop CC 2018, https://www.adobe.com/cn/products/photoshop.html/).

In this paper, we report a singly resonate IC-OPO based on multichannel PPLN. The OPO is pumped by a 1064 nm Nd:YVO_4_ laser, with a nested cavity configuration. The schematic of the IC-OPO is shown in Fig. 1. The 1064 nm laser cavity was formed by the front face of Nd:YVO_4_ crystal and the output mirror of OPO, and the Nd:YVO_4_ crystal was pumped by 808 nm LD from the front face. The input mirror of OPO was in the laser cavity and followed by a multichannel PPLN. The output laser was filtered by two dichroic mirrors, which were transparent to MIR and reflect 808 nm, 1064 nm and near-infrared. The scale of total set-up was 120 mm in length from LD to the output window of OPO. At LD power of 9.1 W, the IC-OPO generated 1.08 W of the maximum idler power at 3.189 μm, indicating conversion efficiency of 11.88% from LD to mid-infrared. By changing channels and temperature of PPLN, the output mid-infrared of IC-OPO was continuously tunable from 2.25 to 4.794 μm. A compact prototype was also fabricated with dimension of 145 × 85 × 42.5 mm^3^ and the weight of less than 2 kg. The power stability was measured to be below 3% RMS tested at the environmental temperature of 40 °C and − 40 °C.

## Methods

### Detailed illustration of experimental set-up

The highest power of LD (Focus Light) was 10 W, whose output theoretically peaked at 808 nm when its operating temperature was at 25–30 °C. The thermal effects could not be ignored when LD pump power was rising. LD was fixed in a water-cooled copper heat sink to relieve the thermal effects. And the refrigerant water had been maintained at the temperature of 25 °C throughout the experiment.

The front face of Nd:YVO_4_ crystal (CASTECH) was HR coated at 1064 nm and AR coated at 808 nm which served as a 1064 nm resonant mirror. Meanwhile IC-OPO output mirror was HR coated at 1064 nm and 1.3–1.8 μm on concave face (R = − 100 mm), HT coated at 2.6–4.1 μm on both sides. These two mirrors formed 1064 nm laser cavity. IC-OPO input mirror was HR coated at 1.4–1.7 μm and HR coated at 2.6–4.1 μm, AR coated at 1064 nm. Focusing lens owned the focal distance of 4.5 mm.

The Nd:YVO_4_ crystal wrapped by indium foil and mounted tightly in a water-cooled copper heat sink was 2 mm × 2 mm × 8 mm in size cooled by refrigerant water. The OPO crystal was 5-mol % MgO:PPLN (CTL Photonics) and was 50 mm long with an aperture of 10 mm × 1 mm. The crystal contained ten grating periods of 27.6–31.6 μm as a nonlinear material in the set-up. PPLN was AR coated at 1.064 μm, 1.4–1.7 μm and 2.6–4.4 μm on both sides. The OPO resonator cavity was packaged in an oven, which was conveniently used to adjust and control the PPLN operating temperature^24^. In addition, this oven was fixed in a three-dimensional adjustment table, used to change channels. The transmittance of IC-OPO input mirror was of 97% at 1064 nm and 0.46–0.058% at signal wave (1500–1700 nm). As for IC-OPO output mirror, its transmittance was of 0.17% at 1064 nm and 0.076–0.13% at signal wave (1400–1700 nm). The two mirrors were separated by 55 mm for linear cavity.

### Results of IC-OPO based on PPLN

**Figure 2:**
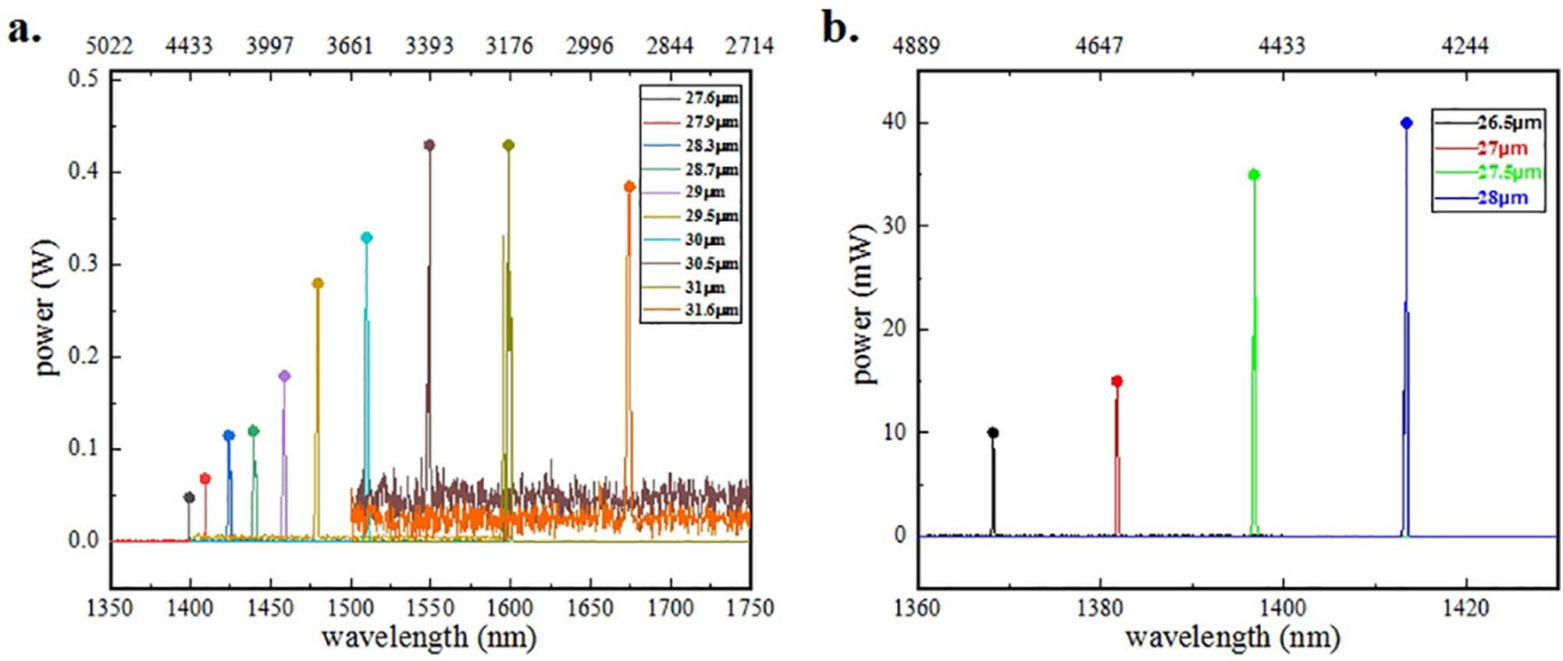
Signal wavelength of different channels. Label of bottom axis means wavelength of signal while top line is corresponding calculated wavelength of idler and the Y-axis is power of idler. (**a**) Shows spectra of PPLN with long period at temperature of 40 °C. (**b**) Shows spectra of PPLN with shorter period at temperature of 45 °C. (Origin 2018, https://www.originlab.com/, Adobe Photoshop CC 2018, https://www.adobe.com/cn/products/photoshop.html/).

We put IC-OPO input mirror and PPLN in laser cavity and adjusted operating temperature as well as channels. Through spectrometer with range of 1200–2400 nm (AQ6375B, YOKOGAWA), wide span of signal light spectra was acquired, which was from 1.40 to 1.67 μm at PPLN operating temperature of 40 °C when LD pump power was 4.84 W, shown in Fig. 2a. Except for wavelength, we also measured power of different idler output while changing the channel of PPLN. The points above each peak mean the power of idler , measured by optical power meter (Thorlabs, series 415c).

According to energy conversion equation in a general three-wave interaction, the idler wavelength can be calculated as

1 $$ \frac{1}{{\lambda_{p} }} = \frac{1}{{\lambda_{s} }} + \frac{1}{{\lambda_{i} }} $$

where λp, λs, λi are the pump, the signal and the idler vacuum wavelength, respectively^25^. Therefore, idler wavelength spans from 2.92 μm to 4.44 μm at operating temperature of 40 °C.

**Figure 3:**
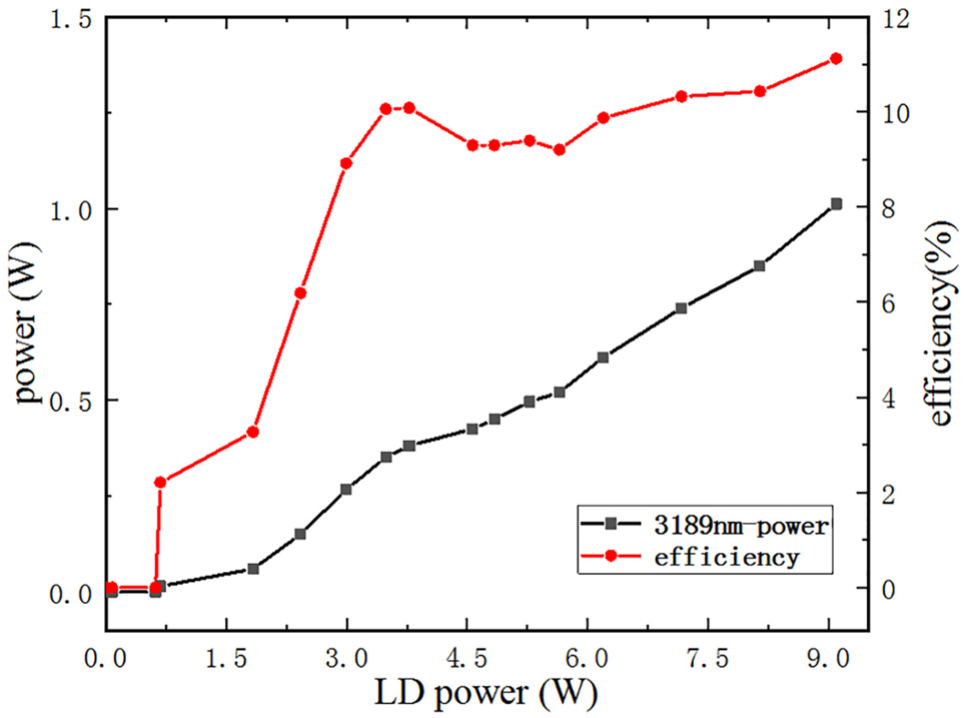
Output power and efficiency versus incident LD power. As the power of LD increase, the output power of idler rises and the efficiency increase except for 4 W. (Origin 2018, https://www.originlab.com/, Adobe Photoshop CC 2018, https://www.adobe.com/cn/products/photoshop.html/).

We increased power from 5 mW to 9.1 W controlling PPLN operating temperature at 40 °C. As for 31 μm, its threshold was found to be 680 mW. When LD pump power was 9.1 W, high power of idler was measured, which was 1.012 W. Efficiency and output power were shown in Fig. 3. The power of rest periods or same period at higher temperature was a little lower. The highest conversion efficiency of 808 nm transferring to idler is 11.88% at 3189 nm. We assumed the efficiency drop was caused by the accumulated thermal-lens effects, which would significantly effect cavity spatial mode when LD was around 4 W.

To broaden the range of MIR output, we tried another multichannel PPLN with 6 shorter grating periods owning dimensions of 33.8 mm × 6 mm × 1 mm and new cavity mirror with wider range of coating film. New IC-OPO output mirror is HR coated at 1064 nm, 1.35–2 μm and AR coated at 2.3–5 μm. New IC-OPO input mirror is HR coated at 1.35–2 μm and HT coated at 2.3–5 μm. Channels of 25.5 μm and 26 μm didn’t generate MIR output shown in Fig. 2b, because span of membrane system coated in OPO cavity has low reflectivity below 1360 nm.

**Figure 4:**
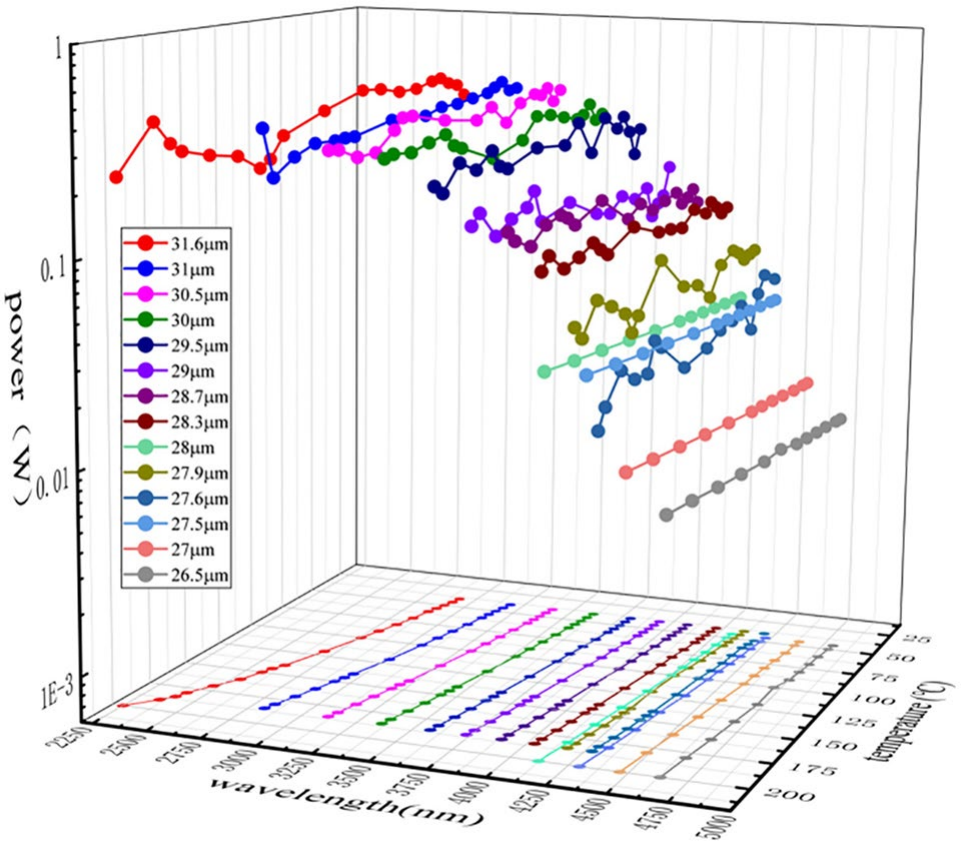
Curves of tuning temperature and periods. Span of idler is from 2.25 to 4.79 μm when pump power is 4.8 W. The temperature is from 40 °C to 200 °C. (Origin 2018, https://www.originlab.com/, Adobe Photoshop CC 2018, https://www.adobe.com/cn/products/photoshop.html/).

We changed temperature from 40 to 200 °C while tuning period from 26.5 to 31.6 μm, measuring wavelength of signal using two multichannel PPLN described above. Wavelength coincided when controlling temperature of adjacent channels shown in Fig. 4. For example, signal wavelength was 1682.98 nm in 31 μm at 160 °C while it’s 1674.29 nm in 31.6 μm at 40 °C. At certain temperature signal wavelength would be the same as that in 31.6 μm at 40 °C. It’s possible to tune output wavelength in a continuous way by varying temperature of crystal or fabricating a crystal with finer grating period increments.

**Figure 5:**
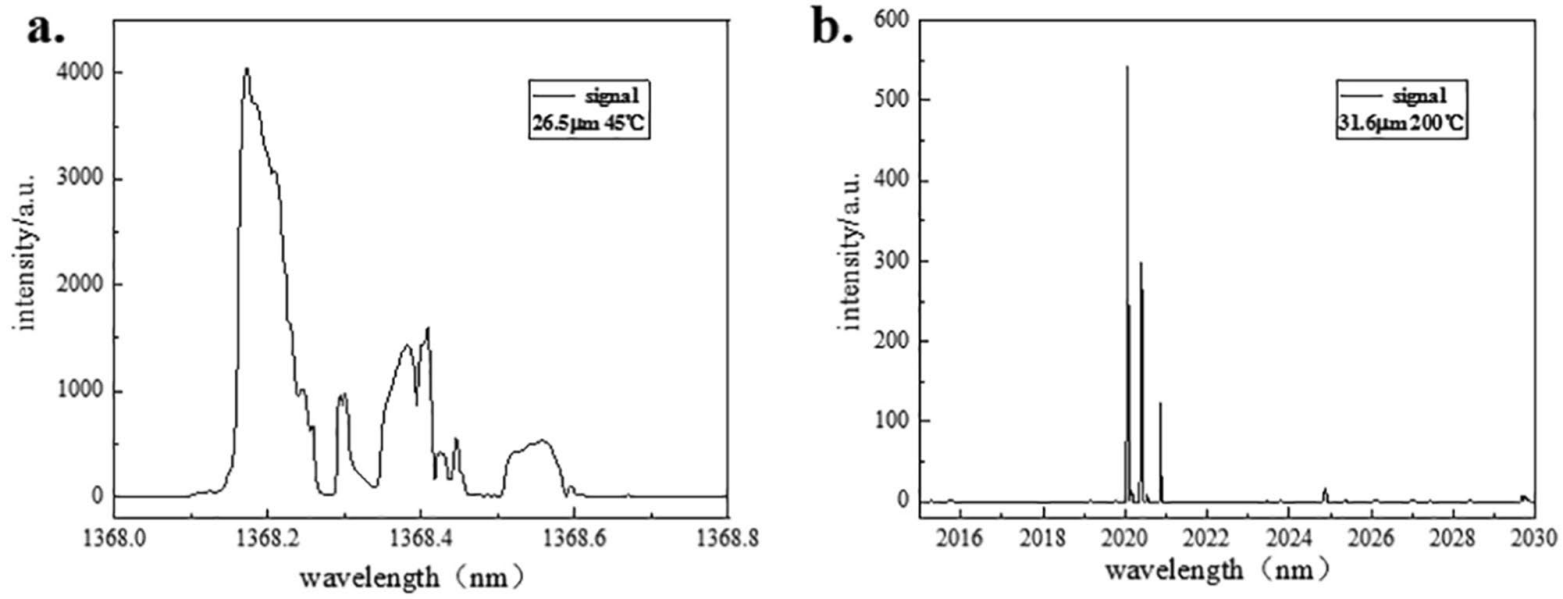
Shortest and longest signal spectra. (**a**) Shows the spectrum of shortest signal; (**b**) shows the spectrum of longest signal. (Origin 2018, https://www.originlab.com/, Adobe Photoshop CC 2018, https://www.adobe.com/cn/products/photoshop.html/).

We measured the shortest signal wave in 26.5 μm at operating temperature of 45 °C when power of LD was 4.8 W. The output spectrum peaked at 1368 nm shown in Fig. 5a. Hence, the longest MIR we calculated was 4793.8 nm. However, the power of 4793.8 nm was very low, which was only 15 mW. From spectra, we could find there existed multiple longitudinal mode. In addition, there was no phenomena of oscillation in 26 μm and 25.5 μm. Consideration is also given to practical factors that limit the OPO performance such as crystal damage mechanism and limits. Equally, wavelength in 26 μm or 25.5 μm is over to edge of 1.35 μm HR membrane. We guess that energy loses because of new PPLN-OPO input mirror without being HR coated at range of idler, which would be improved in our subsequent work.

In 31.6 μm, there was no obvious power loss when we changed temperature from 150 to 200 °C under same condition of pump in 4.8 W. The longest wavelength of signal was obtained which was 2020.058 nm shown in Fig. 5b. Through calculation, wavelength of idler was 2250.139 nm with 230 mW. Instead of running on a single axial mode of the OPO resonator, the cavity tended to run multiple axial modes with relatively wide linewidth. To constraint mode hops, the following experimental configuration should be considered. The thickness of etalon would be about 500 μm which was coated about 50% of reflection at 1064 nm and 1550 nm in both sides. Etalon would be fixed in front of m2, or two Etalons both in laser and OPO cavity.

**Figure 6:**
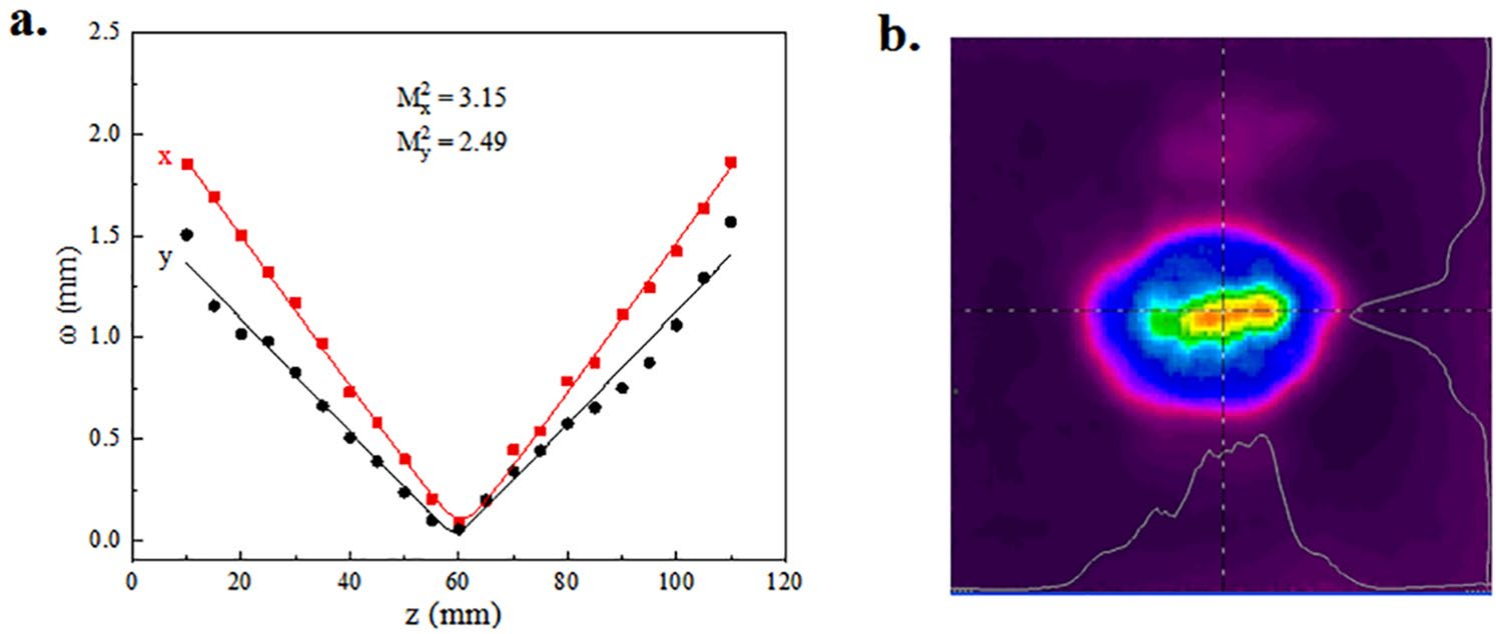
Beam quality of IC-OPO. (**a**) Hyperbola fitting of M^2^ at 3.9 μm. The dots mean measured value while the line is fitting curve. x represents horizontal value while y shows vertical value. (**b**) Intensity distribution of idler beam at 3.9 μm. (Origin 2018, https://www.originlab.com/, pyrocam III control console, pyrocam III control driver, https://www.ophiropt.com/, Adobe Photoshop CC 2018, https://www.adobe.com/cn/products/photoshop.html/).

Horizontal and vertical beam quality of idler whose wavelength was 3.9 μm were measured by the knife-edge method. According to the equation of propagation, we acquired the beam quality.

2 $${\omega^{2} \left( z \right) = \omega_{0}^{2} + \left( {\frac{{M^{2} \lambda }}{{\pi \omega_{0} }}} \right)^{2} (z - z_{0} )^{2} }$$

where ω_0_ is the radius of waist. λ means wavelength of idler. ω(z) is the radius of spot when beam propagate in the distance of z^26^. The results of hyperbola fitting of M^2^ is shown in Fig. 6a. The smaller M^2^ in y direction may be caused by the crystal thickness limitation in y direction. Oscillation with large divergence angles at y direction is suppressed due to the vertical thickness of PPLN. Intensity distribution of idler beam at 3.9 μm is also measured by laser beam diagnostics (Spiricon, pyrocam III), shown in Fig. 6b.

### Configuration of prototype

**Figure 7:**
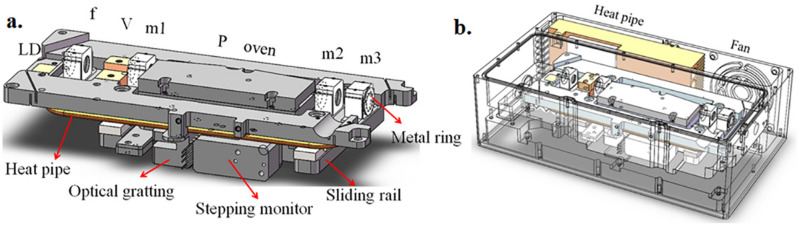
Design of the singly resonant IC-OPO prototype. LD: solid 808 nm laser diode; f: focusing lens; V: Nd:YVO_4_ crystal; m1: IC-OPO input mirror; P: PPLN crystal; m2: IC-OPO output mirror; m3: dichroic mirrors. (Solid Works 2018, https://www.solidworks.com/, Microsoft Office Power Point 2007, https://www.office.com/, Adobe Photoshop CC 2018, https://www.adobe.com/cn/products/photoshop.html/).

Through a series of experiments of IC-OPO, we believed that it was possible to minimize scale of IC-OPO. We designed and assembled the prototype of IC-OPO shown in Fig. 7. We adhered optical mirror to the light path plate. It was difficult to control the amount of adhesive precisely which would influence the stability and efficiency of OPO while adhesive was curing. So, conversion efficiency of prototypes is lower than that of principled set-up which was 8.6% at 3.189 μm. The tuning range is the same as principled set-up.

The prototype was divided into two parts, optical plate and integrated circuit. All optical components were settled in optical plate which was upward side. The integrated circuit was fixed underneath the optical plate. There was aluminum plate to separate these two parts. The optical plate was made of aluminum for heat conduction while the shell of prototypes was made of steel for rigidity. The shape and structure of optical plate were shown in Fig. 7a. In order to improve the precision of adjustment for different channels, we used optical grating, stepping motor and sliding rail which could realize the function of automatic adjustment. In the prototype, the LD couldn’t be fixed in a water-cooled copper heat sink. We used thermo electric cooler (TEC), heat pipe and a fan to dissipate heat and to control the temperature of LD. We also came up with processing small metal rings to fix the cavity mirror. The sample of compact singly resonant IC-OPO was show in Fig. 7b. Our detailed structure of prototypes is still improving. Stepping monitor and temperature controller are both controlled by computer through serial port line 232. The speed of changing tunnel of PPLN was 0.2 mm/s with precision of 17 μm while the temperature changed at the rate of 5 °C/s during heating up. It took longer for decreasing the temperature of PPLN. The total power consumption of the prototype is about 50 W.

## Results and discussion

**Figure 8:**
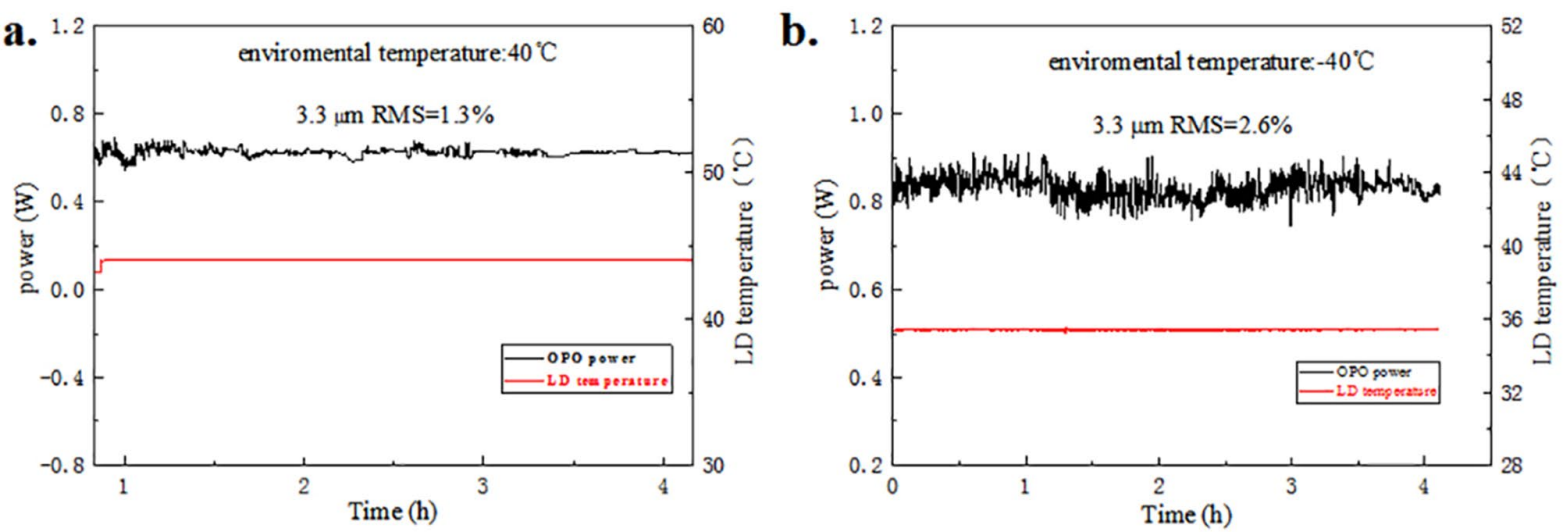
Results of test at low and high temperatures. (**a**) Shows the result at high temperature; (**b**) Shows the result at low temperature. (Origin 2018, https://www.originlab.com/, Adobe Photoshop CC 2018, https://www.adobe.com/cn/products/photoshop.html/).

Our prototype was tested at low and high temperatures while temperature of PPLN was 80 °C shown in Fig. 8. The power of LD was 10 W when a prototype was tested. The average power was 0.624 W when environmental temperature was 40 °C shown in Fig. 8a. The RMS of the MIR output power in 3 h was 1.3%. We could tell that the average power was 0.831 W when environmental temperature was − 40 °C from Fig. 8b. Although its average power was higher, the RMS of the same MIR output was lower. We could know that as environmental temperature changes, the temperature of LD was different, which influenced output of OPO. The power of MIR output would be higher when the temperature of LD was well controlled at 25 °C. As above mentioned, the MIR output exceeded at 1 W when the temperature of LD was at 25 °C. The power was less than 0.8 W when the temperature of LD was at 44 °C while it was over 0.8 W when the temperature of LD was about 34 °C. From the results, we could tell that it was more stable when environmental temperature was higher. We guessed that the working temperature of PPLN was more stable when environmental temperature was higher. Our following work will focus on maintaining inside temperature of prototypes to make sure power of OPO is more stable even environmental temperature is changing.

The volume of this prototype is 145 × 85 × 42.5 mm^3^ and its weight is less than 2 kg. So our prototypes are compact and portable. The performance of prototypes maintains stable after vibration and transport by vehicles without delicate protection. As far as we know, our prototypes are at the leading edge when size, tuning range and efficiency are concerned.

## Conclusion and outlook

In summary, we have presented a compact and widely-continuously tunable MIR singly resonant IC-OPO based on PPLN with multiple grating structures by the means of QPM technique. A spectral tunability from 2.25 to 4.79 μm is demonstrated in the CW region. High power and low threshold are achieved. Conversion efficiency is approximate to 12%. After manufacturing suitable HR mirror and PPLN with specific period, our prototypes are under configuration with good performance. The volume of our prototype is 145 × 85 × 42.5 mm^3^ which weighs less than 2 kg. Through the test at high and low temperature, the power stability at 3.3 μm is measured to be less than 3% RMS. We believe that such a compact, high-efficiency, tunable, stable OPO prototype can be widely used. It has high potential for practical applications. From the spectra of the signal, it is obvious that our singly IC-OPO oscillates in a multiple longitudinal mode. More work is needed to be done to narrow the linewidth of IC-OPO. For example, we could insert Perot-Fabry etalon to constrain the linewidth of 1064 nm and MIR. The design of our prototypes makes it possible to minimize portable IC-OPO without losing its function. If single longitudinal mode was acquired, our prototypes would be more practical.

## Data Availability

The data that support the findings of this study are available from the corresponding author upon reasonable request.
